# Influenza in Relation to the Nervous System

**Published:** 1907-04

**Authors:** 


### INFLUENZA IN RELATION TO NERVOUS SYSTEM.

Collins says that the baneful effects of the pathogenic activity of the Pfeiffer bacillus on the nervous system have been overestimated, and that in reality influenzal and postinfluenzal neurosis and psychoses are not very common. The bacillus of influenza may and does cause meningitis, encephalitis, myelitis and possibly neuritis. The toxins produced by the pathogenic activity of the influenza bacillus are adequate exciting causes of neurasthenia and psychastehnia, of neuritis and neuralgia, of various forms of insanity, of independent nervous systems, such as insomnia, vertigo, headache, and they may be contributory to the causation of any disease that afflicts the human being. Nevertheless, neither the bacillus of Pfeiffer nor its toxins, he declares, is a common cause of nervous disease. The disorders of the nervous system that stand most frequently in causative relation to influenza are those dependent not on the direct action of the bacillus of influenza on the nervous system, but on the activity of toxins produced by the pathogenic activity of these germs. These are neurasthenia and psychasthenia, neuralgia and neuritis, and diseases and disorders of the nervous system attending and dependent on arteriosclerosis.—Med. Record, March 2, 1907.

